# Detecting latitudinal and altitudinal expansion of invasive bamboo *Phyllostachys edulis* and *Phyllostachys bambusoides* (Poaceae) in Japan to project potential habitats under 1.5°C–4.0°C global warming

**DOI:** 10.1002/ece3.3471

**Published:** 2017-10-18

**Authors:** Kohei Takenaka Takano, Kenshi Hibino, Ayaka Numata, Michio Oguro, Masahiro Aiba, Hideo Shiogama, Izuru Takayabu, Tohru Nakashizuka

**Affiliations:** ^1^ Graduate School of Life Sciences Tohoku University Sendai Japan; ^2^ Forestry and Forest Products Research Institute Tsukuba Japan; ^3^ Nagano Environmental Conservation Research Institute Nagano Japan; ^4^ University of Tsukuba Tsukuba Japan; ^5^ Meteorological Research Institute Tsukuba Japan; ^6^ Institute of Industrial Science University of Tokyo Tokyo Japan; ^7^ National Institute for Environmental Studies Tsukuba Japan; ^8^ Research Institute for Humanity and Nature Kyoto Japan

**Keywords:** bioclimatic envelope modeling, invasive plants, non‐hydrostatic regional climate model (NHRCM), RCP8.5 scenario, species distribution modeling, The Paris Agreement

## Abstract

Rapid expansion of exotic bamboos has lowered species diversity in Japan's ecosystems by hampering native plant growth. The invasive potential of bamboo, facilitated by global warming, may also affect other countries with developing bamboo industries. We examined past (1975–1980) and recent (2012) distributions of major exotic bamboos (*Phyllostachys edulis* and *P. bambusoides*) in areas adjacent to 145 weather stations in central and northern Japan. Bamboo stands have been established at 17 sites along the latitudinal and altitudinal distributional limit during the last three decades. Ecological niche modeling indicated that temperature had a strong influence on bamboo distribution. Using mean annual temperature and sun radiation data, we reproduced bamboo distribution (accuracy = 0.93 and AUC (area under the receiver operating characteristic curve) = 0.92). These results infer that exotic bamboo distribution has shifted northward and upslope, in association with recent climate warming. Then, we simulated future climate data and projected the climate change impact on the potential habitat distribution of invasive bamboos under different temperature increases (i.e., 1.5°C, 2.0°C, 3.0°C, and 4.0°C) relative to the preindustrial period. Potential habitats in central and northern Japan were estimated to increase from 35% under the current climate (1980–2000) to 46%–48%, 51%–54%, 61%–67%, and 77%–83% under 1.5°C, 2.0°C, 3.0°C, and 4.0°C warming levels, respectively. These infer that the risk areas can increase by 1.3 times even under a 1.5°C scenario and expand by 2.3 times under a 4.0°C scenario. For sustainable ecosystem management, both mitigation and adaptation are necessary: bamboo planting must be carefully monitored in predicted potential habitats, which covers most of Japan.

## 1 INTRODUCTION

Climate change has caused geographical distribution shifts among many plants (e.g., Lenoir & Svenning, 2015; Parmesan & Yohe, 2003; Walther et al., 2007), including invasive species (Bradley, Blumenthal, Wilcove, & Ziska, 2010), thus threatening numerous ecosystems (WWF, 2016). For example, *Acacia nilotica* (L.) Delile (Fabaceae) has become highly invasive in several parts of the world, and global climate change is likely to increase markedly the plant's potential distribution in Australia (Kriticos, Sutherst, Brown, Adkins, & Maywald, 2003). Therefore, sustainable ecosystem management requires projections on the risk of invasive species expanding their distribution under climate change.

Bamboo is an economically important plant, but some species have the potential to become invasive. A highly versatile plant, bamboo is used for food, building materials, horticulture, paper, textiles, and charcoal, among numerous other applications (FAO, 2010). Since the 1980s, the bamboo industry has developed rapidly in Asia, while also spreading to Africa and the Americas (FAO, 2007). Bamboo forests are estimated to cover a total area of 31.5 million ha and account for about 0.8% of the total global forest area (FAO, 2010).

Bamboo is one of the fastest growing plants on earth (daily growth rate of 30–120 cm), reaching their final height of 5–25 m in a single, 2‐ to 4‐month growing season (He, Cui, Zhang, Duan, & Zeng, 2013). Bamboo growth can have major implications for conservation and environmental management. For example, some researchers expect bamboo to play an important role in climate change mitigation through carbon sequestration (Lobovikov, Schoene, & Lou, 2012; Song et al., 2011).

Because of their rapid growth rate, however, bamboo plants are highly likely to prevent the growth of other plants and decrease species diversity (Chou & Yang, 1982; Larpkern, Moe, & Totland, 2011; Searashi, Maru, Otori, & Nishii, 1989; Yamaguchi & Inoue, 2004; but see Lin et al., 2014). Additionally, the expansion of invasive bamboo forests can change terrestrial water and nutrient cycles (Chiwa, Onozawa, & Otsuki, 2010; Fukushima, Usui, Ogawa, & Tokuchi, 2015; Shinohara & Otsuki, 2015), damage farmlands and artificial forests (Arao, Kondo, & Honma, 2003), as well as increase the risk of sediment disasters (Hiura, Arikawa, & Bahadur, 2004). Global warming will exacerbate these dangers by facilitating distribution shifts of bamboo forests to new areas (Song et al., 2013; Zhang, Liu, Sun, & Wang, 2011).

**Figure 1:**
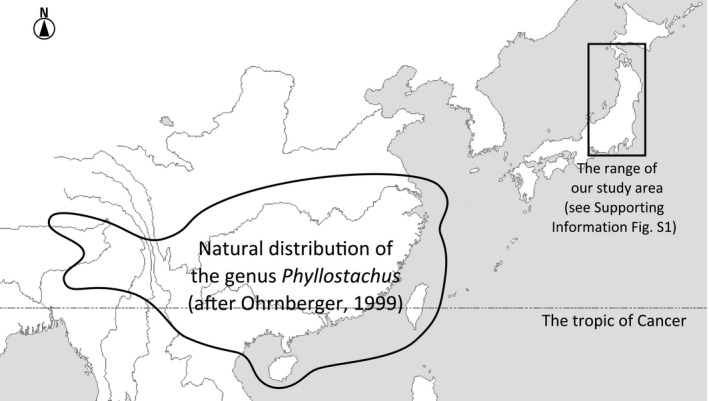
Natural distribution of the genus *Phyllostachus* and the range of our study area

Historically, Japanese people have mainly introduced and used two exotic bamboos (Poaceae), moso (*Phyllostachys edulis* (Carrière) Houzeau de Lehaie) and madake (*P. bambusoides* Siebold et Zuccarini), in managed plantations (See *Study species* Section in Materials and Methods and Figure 1 for details).

Applications include food (mainly for moso), handicraft (mainly for madake) and building materials, horticulture, and protection of houses against wind or fire, as well as river‐wall reinforcement (e.g., Okutomi, 2005b). However, cheaper bamboo imports, heavy labor costs, and the lack of successors to bamboo plantations caused the Japanese bamboo industry to decline in the 1970s (Nakashima, 2001). Consequently, many bamboo plantations have been abandoned and are now left unmanaged, eventually invading the adjacent native vegetation (Nishikawa et al., 2005; Okutomi, Shinoda, & Fukuda, 1996; Suzuki, 2015).

Currently, southern and central Japan are the most affected by invasive bamboos, but anticipated global warming may cause the problem to spread north in the coming decades. Potentially serious risks also exist in other Asian, African, and Latin American countries with burgeoning bamboo industries. For sustainable management of exotic bamboo forests and adjacent ecosystems under global warming, we must understand the growth conditions and how climate change affects potential habitats of major bamboo species.

Someya, Takemura, Miyamoto, and Kamada (2010) conducted the first comprehensive study estimating the distribution probability of exotic bamboo (*Phyllostachys*) forests throughout Japan, in relation to major environmental variables (i.e., climatic elements, soil type, land‐use type, and topography). Their study incorporated GIS data of vegetation maps with other ecological data. The best explanatory variables were warmth index (Kira, 1945), annual precipitation, maximum snow depth, soil type, and land‐use type. A similar study in China (Zhang et al., 2011) also mapped the potential habitats of moso bamboo using presence and pseudo‐absence data, predicting that the potential distribution of moso bamboo will expand up to 266 km northward and increase 13.9% in area by 2070–2099 under SRES A2 (BCCR‐BCM2.0) scenario.

However, both extrapolating occurrence data from vegetation maps and creating pseudo‐absence data increase uncertainty in ecological niche, species distribution, and bioclimatic envelope modeling (cf. Barry & Elith, 2006). For example, although many bamboo forests exist in the northernmost Japanese prefectures (Hokkaido, Aomori, and Iwate) (Suzuki, 1978 and the present study), vegetation maps with a scale of 1:50,000 failed to classify any area in those regions as bamboo forest (Someya et al., 2010). Therefore, to obtain better potential distribution of bamboos, direct observation of presence or absence of bamboo forest is essential.

Moreover, to detect global warming‐related changes, distribution shifts should be monitored together with climatic data in a time series. Previous research analyzed bamboo forest distribution probability on a national scale, but did not consider chronological variation in climatic conditions and bamboo distribution (Someya et al., 2010). Alternatively, chronological changes to bamboo forest expansion were considered, but only on a local (population or landscape) scale (Torii & Isagi, 1997). Therefore, distributional shifts under climate change should be detected through assessments of chronological alteration in actual bamboo occurrence on a regional (i.e., seminational) scale, using accurate meteorological data.

In this study, we directly recorded the presence or absence of bamboo stands near 145 meteorological stations in northern and eastern Japan. Next, we examined aerial photographs from 32–37 years ago to detect chronological distribution shifts (i.e., establishments or colonizations) of bamboo stands. Finally, we fitted bioclimatic models to estimate effects of climatic and land‐use factors on bamboo distribution.

## 2 MATERIALS AND METHODS

### 2.1 Study species

Found throughout tropical, subtropical, and temperate regions, bamboo (subfamily Bambusoideae, family Poaceae) includes over 87 genera and 1,500 species (Bystriakova, Kapos, Stapleton, & Lysenko, 2003; Ohrnberger, 1999).

Six species or forms of the genus *Phyllostachys* are distributed in Japan, all thought to be introduced from China (Ohrnberger, 1999; Figure 1, but see Suzuki, 1978). Among them, we selected the two most common bamboos, moso (*Phyllostachys edulis*) and madake (*P. bambusoides*), as the focal species of this study. The total area of bamboo forest in Japan was 159 000 ha in 2007 (Ministry of Agriculture Forestry and Fisheries of Japan, 2013), comprising 99% moso and madake bamboos at a 3:1 ratio (Torii & Isagi, 1997).

Moso bamboo is the most abundant bamboo species in the world, extensively distributed in southeast and south Asia (Du et al., 2009). Moso bamboo is the most important species in the southern Chinese bamboo industry, and moso plantations exceed 3.8 million ha, representing 70% of the bamboo area in China (FAO, 2007). According to previous studies in China, the primary environmental factors that control moso bamboo distribution and growth are temperature (Xu & Qin, 2003), precipitation (annual range: 800–2,000 mm: Xu & Qin, 2003; Zhou, 1991; but see Gu et al., 2010), and soil conditions (sandy soil with pH 4.5–7.0: Fu, 2001; Jin et al., 2013).

As for madake, a previous study in Japan concluded that its distribution is primarily affected by low air temperatures, although the minimum threshold for madake survival was not determined (Numata, Mitsudera, & Ogawa, 1957).

Moso bamboo was introduced from China to Japan in the 1700s (Suzuki, 1978), whereas madake is thought to be introduced before latter half of the 10th century, which is an estimated period when *Taketori Monogatari* (the Tale of Bamboo Cutter) was written.

Bamboos rely heavily on vegetative growth for the production of new shoots, because flowering is typically a rare event (Janzen, 1976). A flowering interval of moso is 67 years (Watanabe, Ueda, Manabe, & Akai, 1982). Surprisingly, recent genetic analyses imply that moso bamboos of Japan and China (246 samples of 28 wild populations) comprise an identical clone, which is distributed over more than 2,800 km with an estimated biomass of approximately 6.6 × 10^11 ^kg (Isagi et al., 2016). Flowering of madake is rarer (120‐years interval; Janzen, 1976), and most seeds do not germinate (Watanabe et al., 1982). Thus, moso and madake bamboo forests in Japan are considered the result of agricultural efforts (Torii & Isagi, 1997; see also Komatsu, Batagin‐Piotto, Brondani, Gonçalves, & De Almeida, 2011) for a few hundred to a thousand years. In agreement with this consideration, the exotic bamboos are not found in areas that have never been inhabited by people (TN personal observation). In our study, we assumed that local people continuously distributed exotic bamboos as necessity of life (i.e., food and timber) in each locality and that the bamboos colonized successfully under favorable environmental conditions. Therefore, it seems that moso and madake distributions are close to equilibrium with its environment in human habitats.

### 2.2 Study sites and field survey

To obtain a sufficient range of climatic variable and altitude range, we selected 145 AMeDAS (Automated Meteorological Data Acquisition System) stations installed by the Japan Meteorological Agency in northern and eastern Japan, at approximately 35.3–41.5°N and 137.6–142.0°E (Fig. S1). The altitudinal and latitudinal distribution limits of moso and madake bamboos fall within this area (Suzuki, 1978).

In 2012, we drove along with roads and directly surveyed the presence or absence of moso and madake bamboos within a 5‐km radius from each of the 145 AMeDAS stations. To improve efficiency of field observation, we first searched the bamboos within a 1‐km radius from each of the 145 AMeDAS stations then continued searching within 5‐km radius from the station only if we could not find any bamboo stands within the 1‐km radius. We excluded areas where the altitude was 50 m higher or lower than each station so that all of our sampled locations would have similar climate conditions to those observed in the AMeDAS station. If we found a bamboo stand, we identified the species (moso, madake, or both). In the following analyses, we treated data equally irrespective of the sampling effort (i.e., 1‐km or 5‐km radius).

Distribution patterns of moso and madake were similar, except for a slight difference in their northern limits (Fig. S1). Someya et al. (2010) also analyzed the distribution of bamboo forests and reported little difference between moso distribution range and the integrated distribution range of moso, madake, and hachiku (*Phyllostachys nigra* var. *henonis* (Mitford) Stapf ex Rendle) bamboos. Moreover, moso and madake equally depend on their dispersal on human. In addition, we could not run random forest and boosted regression trees (see *Modeling methods considered* section) with less than 100 samples in our dataset. Therefore, we combined the data for moso and madake bamboos in the following analyses.

### 2.3 Analysis of aerial photographs

To detect past distribution of bamboo stands during the last three decades, we investigated historical aerial photographs (116 sites) taken in 1975–1980 at the locations where we found bamboo stands in 2012. We also confirmed the accuracy of recent aerial photographs (2002–2012) through comparisons with our direct observations (i.e., ground‐truth). We could not investigate photographs at seven sites due to time and resource limitations. Thus, we compared 109 bamboo sites in 2012 to their historical photographs. Additionally, we assumed that the 29 sites without bamboo stands in 2012 remained that way during the past three decades, a fundamental limitation of this study.

### 2.4 Environmental variables

#### 2.4.1 Climatic variables considered

Beginning in 1974, AMeDAS measures surface air temperature (1.5 m height), precipitation, hours of sunshine, as well as wind speed and direction at about 1,300 sites in Japan. We obtained positional information (i.e., latitude, longitude, and altitude) and meteorological data for 145 AMeDAS stations from the Japan Meteorological Agency (http://www.data.jma.go.jp/obd/stats/etrn/index). Because post‐1979 data are available at most AMeDAS sites, we calculated separate averages of climatic variables for 1979–1988 (corresponding to bamboo distribution during 1975–1980, from historical aerial photographs) and 2002–2011 (corresponding to 2012, or current, bamboo distribution). These variables were as follows: mean annual air temperature, minimum and maximum air temperatures per year (°C), annual precipitation (mm), growing season precipitation (i.e., during months with mean temperature ≥5°C), nongrowing season precipitation (<5°C), the warmth index (WI: Kira, 1945), and the coldness index (CI: Kira, 1949). Mean annual temperature and minimum and maximum temperatures per year are the mean, minimum, and maximum among temperatures at 00 minute of every hour of everyday in a year, respectively. Therefore, minimum and maximum temperatures per year represent minimum or maximum temperatures of a certain day in each year, respectively.

The warmth index is the annual sum of positive differences between mean monthly temperature and +5°C. It provides a measure of effective heat quantity requisite for plant growth. The coldness index is the annual sum of negative differences between monthly mean temperature and +5°C. Sun radiation data (shortwave radiation, MJ m^−2^ day^−1^) were also obtained from the Mesh Meteorological Dataset (Seino, 1993).

#### 2.4.2 Land cover and land use

We obtained land‐cover and land‐use data from the Second to Fifth Basic Survey on Natural Environment Conservation (surveyed in 1994–1998 with a scale of 1:50,000 provided as vector data [Shapefile]) by the Japanese Ministry of the Environment (http://gis.biodic.go.jp/webgis/sc-023.html). We then used ArcGIS version 10.1 (ESRI, Redlands, CA, USA) to calculate ratios of the forest, farmland, and building areas within a 1‐km radius from each AMeDAS station. Although we measured presence or absence of the bamboos within 5‐km radius from the stations, proportion of areas with different altitude to the station, which we excluded from the studied area of field observation, may increase with distance from the station. Therefore, we used the ratios within 1‐km radius for the index of land cover and land use around the stations. We followed Ogawa et al. (2013) for land‐cover and land‐use classifications.

Because we could not obtain land‐use data for the various time periods assessed, we used the dataset collected in the current study for distribution modeling (response variable: 2012 bamboo presence/absence; explanatory variables: 2002–2011 AMeDAS data and land‐use data).

### 2.5 Ecological niche modeling

#### 2.5.1 Parameter selection

Temperature‐related variables (mean annual air temperature, minimum/maximum air temperature, WI, and CI) were strongly collinear, as were the precipitation variables (annual precipitation and growing season precipitation) (Fig. S2). Collinearity is a severe problem when a model uses data from one time to predict patterns from another period with different or unknown collinearity structure (Dormann et al., 2013).

To select less‐collinear parameters, we ran 10 global (full) logistic regression models (bamboo presence/absence ~ *i *+ *j *+ nongrowing season precipitation + sun radiation + ratio of forest area + ratio of farmland area, where *i* = any of the five temperature‐related variables and *j* = either of the annual precipitation or growing season precipitation). Then, we compared the AICc (corrected Akaike Information Criterion, Hurvich & Tsai, 1989) of all nested models generated with all possible parameter combinations through the *dredge* function from the *MuMIn* package version 1.15.6 (Barton, 2016) in R. Based on this comparison, we chose four candidate parameter sets in the subsequent analysis of modeling methods: 1. mean annual temperature, 2. mean annual temperature + sun radiation, 3. mean annual temperature + sun radiation + precipitation in growing season, and 4. mean annual temperature + sun radiation + precipitation in growing season + ratio of forest area + ratio of farmland area.

#### 2.5.2 Modeling methods considered

Ecological niche modeling uses an increasingly large variety of statistical methods (Franklin, 2009), but few generalizations and recommendations have been provided due to confounding effects of statistical methods, species traits, data characteristics, and accuracy metrics across studies (Miller, 2014). A test of generalized linear models (GLMs), random forest (Breiman, 2001), boosted regression tree (BRT; Elith, Leathwick, & Hastie, 2008), maximum entropy (MaxEnt; Phillips & Dudík, 2008), and genetic algorithm for rule‐set prediction (GARP; Stockwell et al., 2006) revealed that all five performed well even when the species–environment relationship was nonlinear (Elith & Graham, 2009). However, GARP did not handle categorical predictors or interactions between predictors well (Elith & Graham, 2009). Given that these methods have advantages and disadvantages in various contexts, more than two modeling methods should be applied to the same dataset during analysis (Franklin, 2009).

We therefore compared the performances of four modeling methods (logistic regression, generalized additive models (GAMs; Guisan, Edwards, & Hastie, 2002), random forest, and BRT) in predicting current bamboo distribution, using four candidate parameter sets (see *Parameter selection* section). A logistic regression is a GLM with a binomial family (logit link) and has the advantage of possessing easily interpretable predictor coefficients (Franklin, 2009). In contrast, predictor coefficients are difficult to interpret in GAMs, but the method offers a flexible and automated approach to describing nonlinear relationships between predictors and response variables (Franklin, 2009). Random forest and BRT are machine‐learning techniques that assemble numerous relatively independent decision trees. Both automatically identify interactions between variables, whereas interactions must be specified in advance for GLMs and GAMs (Franklin, 2009). However, the latter two models are better suited to reflect theoretical findings on the shape and nature of a species’ response (or realized niche). Overall, a trade‐off exists between predictability (random forest and BRT) versus interpretability (GLMs and GAMs). Models were implemented in R version 3.3.2 (R Core Team, 2016). Detailed settings are described in Data S1.

#### 2.5.3 Model validation and selection

We compared predictabilities of all possible combinations between the four parameter sets and the four modeling methods. Leave‐one‐out cross‐validation (LOOCV) was conducted for each combination, and a threshold value of bamboo existence was determined with the *coords* function from the *pROC* package (Robin et al., 2011) in R. To achieve better handling of our imbalanced dataset (116 presences and 29 absences), we used Youden's J statistics (sensitivity + specificity: Youden, 1950) for choosing a threshold to classify bamboo presence and absence because usage of threshold‐dependent measures of accuracy and determination of appropriate threshold mitigate issues related with biased, imbalanced, or skewed data (Franklin, 2009).

Predictability indices were then calculated, including area under the receiver operating characteristic curve (AUC; Swets, 1973), Matthews correlation coefficient (MCC; Matthews, 1975), informedness (sensitivity + specificity − 1; Powers, 2011), accuracy ([true positives + true negatives]/total n), sensitivity (true positives/[true positives + false negatives]), specificity (true negatives/[false positives + true negatives]), positive predictive value (true positives/[true positives + false positives]), and negative predictive value (true negatives/[false negatives + true negatives]).

Finally, we selected a model for projection. We accounted for comparability with future studies by considering the predictability and availability of parameters in general climate models (such as the Coupled Model Intercomparison Project Phase 5 [CMIP5]; Taylor et al., 2012).

#### 2.5.4 Current and future climate simulations

As boundary conditions of regional climate simulations, we used present and future global climate simulations from the Meteorological Research Institute (MRI) atmospheric global climate model (AGCM), with approximately 20 km horizontal resolution (Mizuta et al., 2012). Using the AGCM, we performed climate simulations of current (1979–2003) and future (2075–2099) conditions under the RCP8.5 emission scenario (van Vuuren et al., 2011). Sea surface temperature (SST) distribution critically affects the atmosphere and was set as a lower boundary condition of the AGCM. Therefore, model uncertainty may arise depending on the SST distribution selected. Uncertainty for the current climate simulation was removed with the use of observational SST distribution data from HadISST1 (Rayner et al., 2003). To take into account uncertainties in future SST changes across climate models, and to reduce computing costs, four future SST distributions were selected to construct a four‐member ensemble experiment (Mizuta et al., 2014). One SST distribution was taken from the ensemble average of CMIP5 atmosphere–ocean model (AOGCM) RCP8.5 simulations, while the remaining SST distributions were derived from a cluster analysis of CMIP5 AOGCMs’ SST change patterns (Mizuta et al., 2014). These four SST distributions covered the full range of projection uncertainty with a small number of experiments that did not require using every CMIP5 SST distribution.

We dynamically downscaled AGCM global climate simulations to a 5‐km horizontal resolution in the region around Japan (see Fig. S3 for the domain), using MRI's nonhydrostatic regional climate model (NHRCM) (Murata 2015) during current (September 1980–August 2000) and future (September 2076–August 2096) periods.

We used temperature and sun radiation outputs, two explanatory variables in our distribution modeling (see *Parameter selection* section). These two variables were averaged annually and over 20 simulation years. As model incompleteness inevitably leads to bias even with decreased uncertainties in the current climate model, we performed bias correction (see Data S2) to obtain more realistic results.

[Correction added on 10 November 2017 after first online publication: Figure 1 and Supporting information Fig. S3 in Section 2.5.4 have been revised in this version.]

#### 2.5.5 Climate scenarios under various global warming levels

To inform climate policies, impact assessments under different global warming conditions are important, and many previous studies have focused on increases between 2°C and 4°C (e.g., Table 1 of Assessment Box SPM.2 in IPCC, 2014). However, after the Paris Agreement in the United Nations Framework Convention on Climate Change (UNFCCC), which pursue efforts to limit warming to 1.5°C, climate change impacts under the 1.5°C warming level are getting more attention. Therefore, we projected climate change impacts under 1.5°C, 2.0°C, 3.0°C, and 4.0°C increments relative to the preindustrial period.

We applied pattern scaling (e.g., Ishizaki et al., 2012; Wigley, Raper, Smith, & Hulme, 2000) to produce climate scenarios for the four global warming levels (Fig. S6):

1. We calculated spatial patterns in 20‐year mean differences between the bias‐corrected outputs of the present and future climate regional NHRCM simulations.
2. The global mean temperature differences between the present and future MRI AGCM climate simulations were 3.49, 3.57, 3.50, and 3.41°C for ensemble mean SST and SST clusters 1–3, respectively. The regional spatial patterns calculated in step (1) were divided by the global mean temperature differences to obtain a “scaling pattern,” or climate variation per 1°C global warming.
3. We calculated 20‐year running mean of globally and annually (from September to August) averaged temperature using historical and RCP8.5 future simulations (September 1980–August 2100) of the 34 CMIP5 models. Next, we computed the multimodel mean for each running mean. The anomaly relative to the value in 1990 (1980–2000 average) was obtained in each year from 1990 to 2090, which represented the degree of the progress of global warming. It should be noted that the global mean observed temperature on September 1980–August 2000 was already 0.5°C above to the preindustrial condition.
4. To obtain climate scenarios of X°C global warming relative to the preindustrial condition, we inflated the scaling pattern of step (2) by “X°C−0.5°C” and added the result to the bias‐corrected outputs of present climate NHRCM simulations.
5. To investigate the year when specified global warming levels will be exceeded, we used the 20‐year running global mean temperature anomaly from step (3). Note that the year is an estimation according to the ensemble mean of CMIP5 models.

#### 2.5.6 Prediction of bamboo distribution

To evaluate how climate change will influence bamboo distribution, we mapped probabilities of potential bamboo habitats using climate scenarios under the four global warming levels, then compared the ratio of potential habitat areas in central and northern Japan (north of 35°N and east of 136°E). Although our model was fit in Honshu Island, and ecosystem of Honshu and that of Hokkaido are different, we extrapolated our model to Hokkaido Island. It is because motivation of local people to plant and eat bamboos still exist regarding from our interviews to local people through fieldworks, and because it is meaningful to predict northward expansion of potential habitats for sustainable ecosystem management. However, extrapolation could produce ecologically inappropriate predictions (Elith, Kearney, & Phillips, 2010). To evaluate the extent of extrapolation, we also calculated the multivariate environmental similarity surface (MESS, Elith et al., 2010) with some modifications (see Data S3 for details).

## 3 RESULTS

### 3.1 Geographical distribution and chronological change in bamboo stands

**Figure 2:**
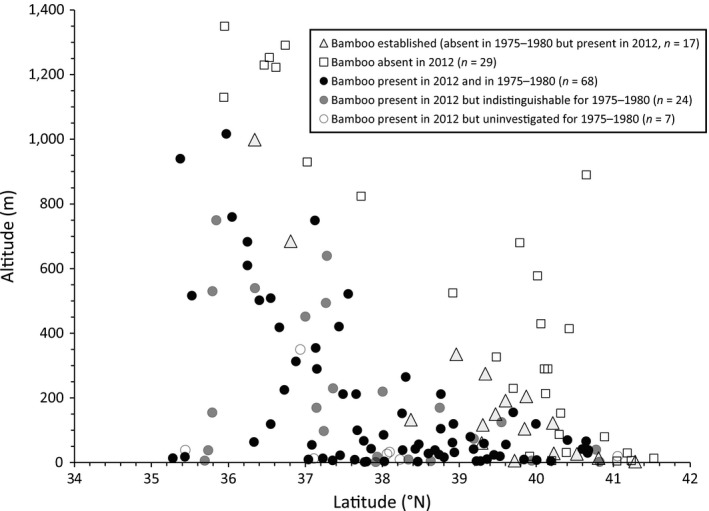
Latitudinal and altitudinal distribution of study sites with presence, absence, or new establishment of bamboo stands

Bamboo stands were found at 116 of 145 study sites in the 2012 field survey. Among them, moso and madake bamboos were present at 104 and 73 sites, respectively (Fig. S1). The distributional limits followed a latitudinal and altitudinal gradient (Figure 2).

At 17 of 109 investigated sites with historical aerial photographs, bamboo stands were absent and therefore considered established post‐1975 (Figure 2 and Fig. S1). These 17 sites were located along the latitudinal and altitudinal limit of bamboo distribution (Figure 2). In the historical photographs, the presence or absence of bamboo stands could be confirmed at 68 sites, but indistinguishable at 24 sites (Figure 2).

### 3.2 Climatic limits on bamboo distribution

Bamboo stands were not found at study sites where 1) mean annual maximum temperature was below 28.8°C, 2) mean annual temperature was below 8.6°C, and 3) mean annual minimum temperature was below −16.8°C (Fig. S4). Our data generally agree with previous research (Fu, 2001) stating that moso bamboo cannot withstand temperatures around or below −18 to −20°C. We also did not find bamboo stands at study sites where CI was below −27.9 and WI was below 68.1, slightly lower than previous reports (Someya et al., 2010) of Japanese *Phyllostachys* bamboos in areas with a WI range of 72–205.

All temperature‐related variables (averages of mean annual temperatures, annual maximum/minimum temperatures, WI, and CI) generally increased between 1979–1988 and 2002–2011, whereas sun radiation declined (Fig. S4) presumably because of increment of cloud. Annual precipitation did not show a clear trend. The means of temperature‐related variables were highest in the “bamboo present” sites, followed by “bamboo established” sites and then “bamboo absent” sites (Fig. S4). Thus, the increasingly warmer climate over the last three decades has spurred bamboo growth in new sites (“bamboo established”) along latitudinal and altitudinal limits of bamboo distribution.

### 3.3 Ecological niche modeling

#### 3.3.1 Parameter selection

The best 10 GLMs, in terms of AICc, are summarized in Table S1. The parameter combination of the minimum‐AICc model was [WI + sun radiation] followed by [maximum temperature of a year + annual precipitation + sun radiation], [maximum temperature of a year + sun radiation], and [mean annual temperature + sun radiation]. Delta AICc values were small (<0.64) among these models. The fourth model has the highest MCC (0.78), whereas the third model has the highest AUC (0.939).

Temperature‐related variables were positively and significantly (*p *<* *.001) correlated with bamboo distribution. Sun radiation also showed positive and significant (*p *<* *.05) correlation. Effect sizes (z‐values) were greatest for temperature and high for sun radiation, but far lower for precipitation and land‐use types.

Among the temperature‐related variables, we selected mean annual temperature as the representative predictor for comparability with future studies, based on parameter availability in general climate models.

#### 3.3.2 Selected model

We further compared predictability among different modeling methods in combination with different sets of explanatory variables (Table S2). The two explanatory variables GLM [mean annual temperature + sun radiation] had the highest MCC (0.780), informedness (0.759), accuracy (0.931), and AUC (0.922). Thus, this model was used for subsequent analyses. Incorporation of growing season precipitation and forest to farmland ratios did not improve predictability.

**Figure 3:**
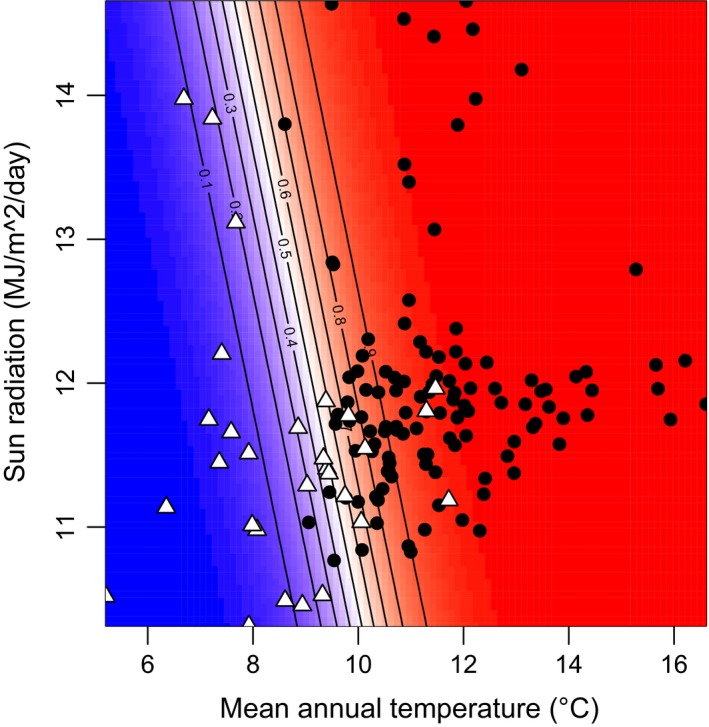
Isothermal graph showing probability as potential habitat of bamboos along mean annual temperature and sun radiation predicted by the best GLM. Contours and colors (blue to red) indicate probability (0 to 1). Points are plotted according to actual data of each study site in 2002−2011. Black circles and white triangles indicate presence or absence of bamboos in 2012, respectively

Bamboo presence and absence were separated with the optimal threshold value (0.606) (Table S2). The GLM predicted that the probability of potential bamboo habitats rapidly increases under mean annual temperatures of 7°C to 11°C, with sun radiation exerting a secondary effect (Figure 3).

### 3.4 Projection of current and future bamboo distribution

#### 3.4.1 The extent of extrapolation

Modified version of the multivariate environmental similarity surface (MESS, Elith et al., 2010), or dissimilarity, showed the extent of extrapolation from the reference points (i.e., 145 AMeDAS stations in 2002–2011) to different space (north of 35°N and east of 136°E) and time (1980–2000 and 2075–2095) (Data S3).

In current temperature (upper left panel of Data S3), rather many high‐altitudinal areas of both Honshu and Hokkaido Islands showed extrapolation with lower temperature. In future temperature (lower left), most of area was included in the range of reference points except for area with higher and lower extreme of altitude and latitude. Overall, the extent of extrapolation was limited in future prediction, whereas the extent of extrapolation was larger in current prediction toward lower temperature.

#### 3.4.2 Impacts of different warming levels

**Figure 4:**
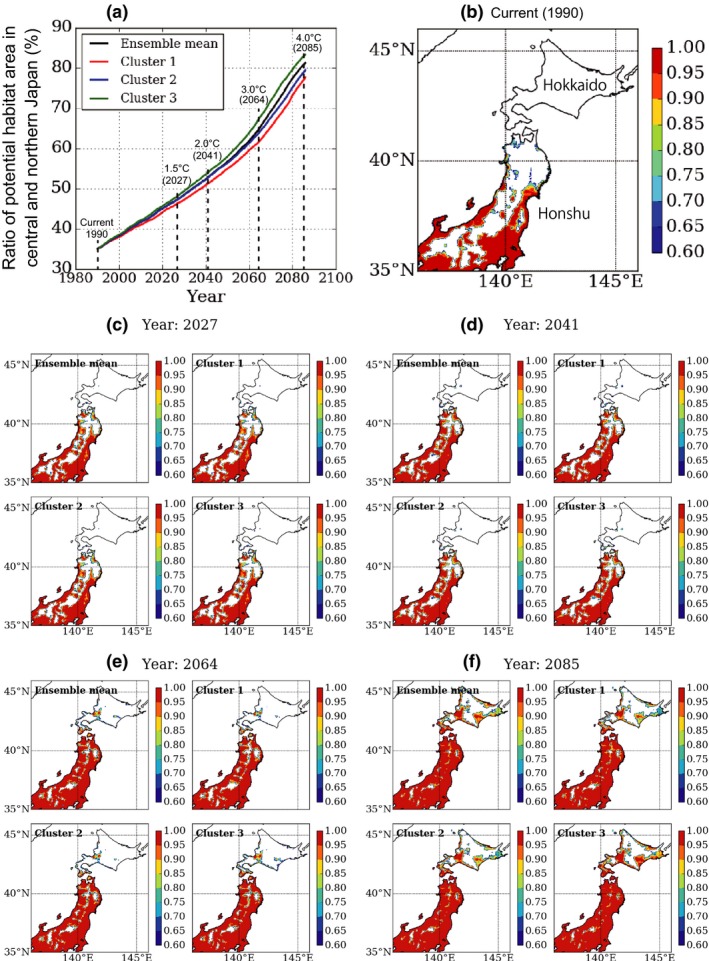
Projection of potential habitats for moso and madake bamboos in central and northern Japan during current (1990) and future (2027, 2041, 2064, and 2085) periods. Four sea surface temperature distributions (i.e., ensemble mean and clusters 1–3) under the RCP 8.5 scenario were considered in future projections (see the text for details). Each listed year represents a 20‐year running mean. Years 2027, 2041, 2064, and 2085 represent mean global warming levels of 1.5°C, 2.0°C, 3.0°C, and 4.0°C above preindustrial conditions, respectively. (a) Ratio of potential habitat area. (b‐f) Geographical distributions. Colors indicate probability as a potential habitat predicted by a generalized linear model. Note that the threshold value for separating bamboo presence or absence is 0.606

Projected changes in mean annual temperature and sun radiation between 1980–2000 and 2076–2096 are shown in Fig. S5. Under the current climate, potential bamboo habitats were projected to account for 35.0% of the Japanese land area north of 35°N and east of 136°E (Figure 4a). However, bamboo distribution remained south of 41°N, in areas that included plains and hilly regions of central Honshu, as well as the coasts of northern Honshu (Figure 4a, b).

The RCP8.5 scenario estimated that global warming levels of 1.5°C, 2.0°C, 3.0°C, and 4.0°C would be exceeded by 2027, 2041, 2064, and 2085 (middle of the 20‐year period), respectively (Figure 4a). Under 1.5°C, potential bamboo habitats were predicted to reach 43.25°N, expanding 240 km northward (Figure 4c) and accounting for 46.3%–48.0% of the focal area (range of the four SST distributions, see Materials and Methods) (Figure 4a). Under 2.0°C, potential habitats reached 44.3°N, expanding 370 km northward (Figure 4d) and accounting for 51.2%–53.9% of the focal area (Figure 4a). Under 3.0°C, potential habitats reached 44.4°N, expanding 380 km northward (Figure 4e) and accounting for 61.2%–66.8% of the focal area (Figure 4a). Finally, under 4.0°C, the potential habitat expanded 500 km northward to cover all of Honshu, reaching the northern tip of Hokkaido Island (45.5°N) but excluding its alpine and subalpine zones (Figure 4f), accounting for 76.8%–83.2% of the focal area (Figure 4a). We created map animations to better visualize the transition from current habitats to future projections (Video S1).

## 4 DISCUSSION

We confirmed that exotic bamboos are widely distributed in northern and eastern Japan, whereas Someya et al. (2010) estimated presence probabilities in this area to be much lower than 0.5. We further detected bamboo distribution shifts, evaluated important environmental factors, and concluded that the shifts were associated with a warming climate.

### 4.1 Environmental factors limiting bamboo distribution

Moso and madake bamboo distribution in northern Japan depends primarily on temperature, and a simple, two‐parameter (mean annual temperature and sun radiation) GLM successfully predicted current bamboo distribution with 0.931 accuracy. Further, even a single‐parameter GLM with only mean annual temperature explained bamboo distribution with 0.923 accuracy.

Previous research indicated that minimum temperature in a given year restricts madake and moso bamboo distribution (Fu, 2001; Numata et al., 1957). However, we found that minimum temperature and CI were not included in any of the 10 best (minimum AICc) models, whereas mean annual temperature, annual maximum temperature, and WI were. Further eco‐physiological studies are therefore necessary to determine how temperature conditions restrict colonization and development of major exotic bamboo species in Japan.

Although the dwarf bamboo *Sasamorpha borealis* (Hack.) Nakai (Poaceae), a keystone species in Japanese forests, requires high precipitation during their growing season (May–July) (Tsuyama et al., 2011) even in humid Japanese environments, our models inferred that precipitation played little role in predicting moso and madake distribution. This would be explained by differences in characteristics among bamboo species. On the other hand, lower annual precipitation (<800 mm) in southern China restricts moso distribution, with spring droughts adversely affecting moso bamboo shooting (Fu, 2001). Because annual precipitation exceeded 930 mm at all sites of the present study, precipitation likely becomes an important factor only in drier regions.

Most exotic bamboo forests in Japan originated from human transplantation (Okutomi, 2005a), a notable limitation in our study's aim to understand bamboo distribution. When modeling the distribution of such semi‐naturalized organisms, resultant distribution patterns may represent the intensity of artificial introduction rather than natural expansion, confounding the ability to draw conclusions. Thus, the history and intensity of bamboo introduction should be explicitly incorporated in future models (Guisan & Thuiller, 2005).

Our models suggested that the ratios of forests and farmland had little explanatory power for the bamboo distribution in our dataset, at odds with a previous model that selected forest, farmland, and building‐area ratios as explanatory variables (Someya et al., 2010). This difference appears at least partially due to sample sizes (*n *=* *244 327 in Someya et al., 2010 and *n *=* *146 in the present study). Moreover, our study sites were selected within a 5‐km radius from the AMeDAS stations, which are generally located at accessible human inhabited area such as roadsides at elevations below 1 500 m for its operation and maintenance, but not in a deep forest. Such sampling design of the present study may have restricted the land‐use types recorded and limited the detection of their effects on bamboo distribution.

Finally, several variables appear to affect bamboo distribution that was not examined in our models. First, soil pH and texture restrict moso bamboo distribution in China, along with precipitation (Fu, 2001; Jin et al., 2013). Further, bamboos are likely to have soil preferences; madake appears to prefer sandy loam (Numata et al., 1957), and surface geology was selected as an explanatory variable in the AIC minimum model of Someya et al. (2010). Future, more comprehensive models of bamboo distribution should consider all of these variables.

### 4.2 Bamboo distribution projections and implications for sustainable ecosystem management

The distribution limit of bamboo was predicted to expand 500 km northward by 2085, at maximum. Additionally, potential habitats in central and northern Japan were expected to increase from 35% (1980–2000) to 46%–48% (under 1.5°C global warming), 51%–54% (2.0°C), 61%–67% (3.0°C), and 77%–83% (4.0°C). Therefore, under the most conservative global warming estimate, the potential habitat will enlarge by 1.5 times, while expanding by 2.3 times under the highest warming scenario.

These values are more or less comparable with previous predictions (Zhang et al., 2011) that the potential distribution of moso bamboo in China will expand 266 km northward and increase 13.9% in area by 2070–2099 under SRES A2 (BCCR‐BCM2.0) scenario. Together, the data indicate that the 1.5°C scenario is clearly preferable to the 4.0°C scenario (i.e., RCP8.5) in terms of managing the invasive expansion of exotic bamboos. Therefore, efforts aimed at limiting future warming to 1.5°C should be beneficial.

Analyses above do not necessarily mean that the affected areas will be completely covered with moso and madake, but just predict potential habitats. We also must keep in mind various uncertainty in species distribution modeling. Because current distribution (1980–2000) was predicted with slightly extrapolated lower temperatures, it might result in underestimation (i.e., overestimation of expansion rate toward future). On the other hand, we assumed that the exotic bamboo distribution is close to equilibrium with its environment. Violation of this assumption would result in underestimation of potential habitat around its northern limit.

Overall, global warming definitely increases the success rate of colonization by these highly invasive species. Once a bamboo plantation is abandoned, it poses a serious risk of invading surrounding ecosystems (Suzuki, 2015). In 2015, Ministry of the Environment and Ministry of Agriculture, Forestry and Fisheries of Japanese government designated bamboos of the genus *Phyllostachys* as concerned alien species that are industrially important and that require appropriate management. To better adapt under climate change, improved oversight of bamboo planting and heightened caution from local communities are necessary (Someya et al., 2010). Outside Japan, countries that actively cultivate exotic bamboo will also need to recognize the need for greater regulation and vigilant practices targeted at preventing bamboo invasion (International Network for Bamboo and Rattan (INBAR), 2014).

## CONFLICT OF INTEREST

None declared.

## AUTHOR CONTRIBUTIONS

TN and AN conceived the study and collected bamboo presence/absence data. AN analyzed aerial photographs. AN and KTT downloaded and processed AMeDAS data. MO downloaded land‐cover and land‐use data and KTT processed them. KTT, MO, and MA implemented and validated ecological niche modeling. KH and IT performed climate simulations and bias correction. HS conducted pattern scaling. KH and KTT projected bamboo distribution. KTT, KH, and HS prepared the manuscript and all authors revised it critically.
