# Supplementary material for: Detecting latitudinal and altitudinal expansion of invasive bamboo Phyllostachys edulis and Phyllostachys bambusoides (Poaceae) in Japan to project potential habitats under 1.5°C–4.0°C global warming
Source: Ecol Evol. 2017 Oct 18;7(23):9848–59. doi: 10.1002/ece3.3471 (PMC5723622; doi:10.1002/ece3.3471)
Supplement: Supplementary file 2 [file ECE3-7-9848-s002.pdf]

Supporting Information fig S2. Scatterplot matrices of data for modelling, including bamboo presence or absence in 2012, climate data in 2002–2011, and land-use types

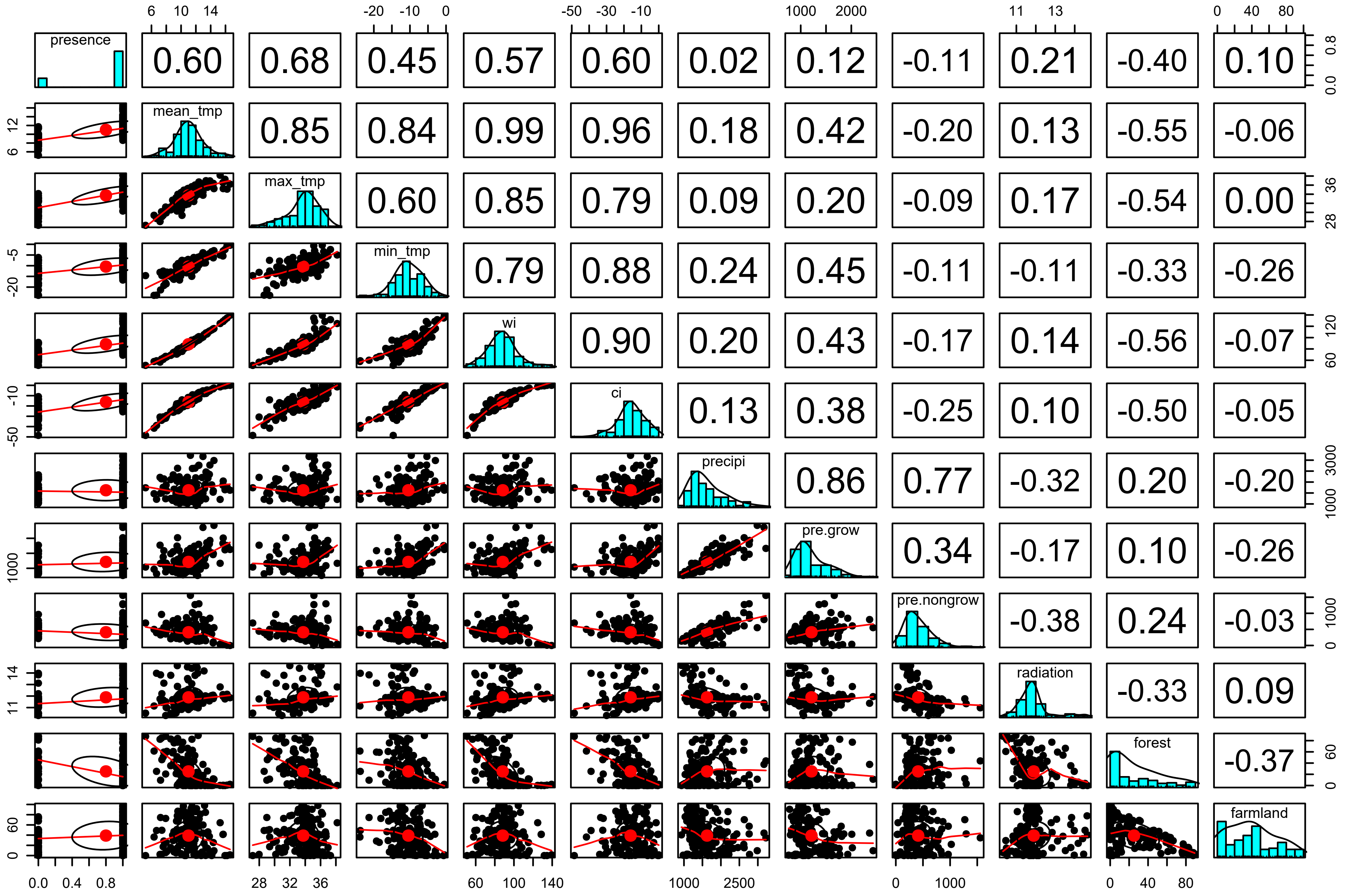

Supporting Information fig S2. Scatterplot matrices of data for modelling, including bamboo presence or absence in 2012, climate data in 2002–2011, and land-use types.

The figure was created with the pairs.panels function of the psych package (Revelle, 2016) in R. Numbers in the right triangular matrix represent the Pearson's correlation coefficient. The histogram of each variable is shown on the diagonal. Scatterplots of each variable pair and the lowest locally fit regression line are shown below the diagonal. Abbreviations: presence, bamboo presence/absence in 2012; mean_tmp, mean annual temperature; max_tmp, maximum temperature per year; min_tmp, minimum temperature per year; wi, the warmth index; ci, the coldness index; precipi, annual precipitation; pre.grow, precipitation during growing season; pre.nongrow, precipitation during non-growing season; radiation, sun radiation; forest, ratio of forest area; farmland, ratio of farmland area.
