# Supplementary material for: Detecting latitudinal and altitudinal expansion of invasive bamboo Phyllostachys edulis and Phyllostachys bambusoides (Poaceae) in Japan to project potential habitats under 1.5°C–4.0°C global warming
Source: Ecol Evol. 2017 Oct 18;7(23):9848–59. doi: 10.1002/ece3.3471 (PMC5723622; doi:10.1002/ece3.3471)
Supplement: Supplementary file 3 [file ECE3-7-9848-s003.docx]

Data S1. Detailed settings in model implementations

We applied *stats* package version 3.3.2 in R (R Core Team, 2016) for generalized linear models (GLMs), *mgcv* package version 1.8-12 (Wood, 2011) for generalized additive models (GAMs), *randomForest* package version 4.6-12 (Liaw & Wiener, 2002) for random forest, and *gbm* package version 2.1.1 (Ridgeway et al, 2015) for boosted regression trees (BRT).

In GLM and GAM, the binomial distribution was applied.

In machine learning, a model fitting is conducted through a process known as model training. The term ‘hyperparameters’ express properties of the model such as its complexity or how fast it should learn in the training.

As hyperparameters of random forest, *mtry* (number of variables randomly sampled as candidates at each split) = 1, 2, …, 6 and *nodesize* (minimum size of terminal nodes) = 2, 3, …, 20 were examined. *ntree* (number of trees) was set to 500.

As hyperparameters of BRT, another machine-learning method (Elith et al., 2008), *interaction.depth* (i.e. tree complexity) = 1, 2, 3; *n.minobsinnode* (i.e. minimum number of observations in the trees’ terminal nodes) = 2, 3, …, 20; and *bag.fraction* (i.e. the fraction of the training set observations randomly selected to propose the next tree in the expansion) = 0.5, 0.75 were examined. *shrinkage* (i.e. learning rate) and *n.trees* (number of trees) were fixed to 0.0005 and 500, respectively.

Hyperparameter optimizations were conducted with grid search, which is an exhaustive searching through all specified subset of the hyperparameters. The Matthews correlation coefficient (MCC: Matthews, 1975) in a leave one out cross validation (LOOCV) was used as a criterion. If there is a tie in MCC, the area under the ROC curve (AUC: Hanley and McNeil, 1982) was used.

Hanley JA, Mcneil BJ (1982) The meaning and use of the area under a receiver operating characteristic (ROC) curve. Radiology*,* **143**, 29-36.

Liaw A, Wiener M (2002) Classification and Regression by randomForest. R News*,* **2**, 18-22.

Matthews BW (1975) Comparison of the predicted and observed secondary structure of T4 phage lysozyme. Biochimica et Biophysica Acta (BBA) - Protein Structure*,* **405**, 442-451.

R Core Team (2016). R: A language and environment for statistical computing. R Foundation for Statistical Computing, Vienna, Austria. URL https://www.R-project.org/.

Ridgeway G with contributions from others (2015). gbm: generalized boosted regression models. R package version 2.1.1. https://CRAN.R-project.org/package=gbm

Wood SN (2011) Fast stable restricted maximum likelihood and marginal likelihood estimation of semiparametric generalized linear models. Journal of the Royal Statistical Society: Series B (Statistical Methodology)*,* **73**, 3-36.
