# Supplementary figures and images for: Detecting latitudinal and altitudinal expansion of invasive bamboo Phyllostachys edulis and Phyllostachys bambusoides (Poaceae) in Japan to project potential habitats under 1.5°C–4.0°C global warming
Source: Ecol Evol. 2017 Oct 18;7(23):9848–59. doi: 10.1002/ece3.3471 (PMC5723622; doi:10.1002/ece3.3471)

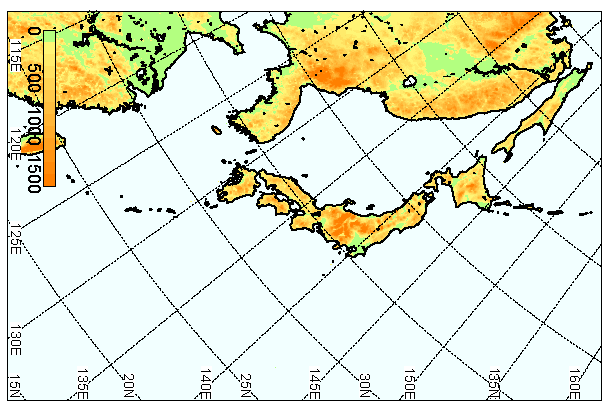

Supplement: Supplementary file 4 [file ECE3-7-9848-s004.png]
