# Supplementary material for: Detecting latitudinal and altitudinal expansion of invasive bamboo Phyllostachys edulis and Phyllostachys bambusoides (Poaceae) in Japan to project potential habitats under 1.5°C–4.0°C global warming
Source: Ecol Evol. 2017 Oct 18;7(23):9848–59. doi: 10.1002/ece3.3471 (PMC5723622; doi:10.1002/ece3.3471)
Supplement: Supplementary file 5 [file ECE3-7-9848-s005.docx]

**Data S2.**

**Description of bias correction methods.**

The Bias correction method makes statistical features of the current climate simulation results identical to those of the observational data. There are various methods of bias corrections. Some of them correct the mean value, others correct the variance, skewness, or kurtosis, and others correct all *n-th* moments. In the present study, we adopted one of the last methods, which matches the cumulative distribution function (CDF) of model results with that of observation (Fig. 1). Given current climate model result $X_{mc}$ yields its percentile (for example, approximately 60% in Fig. A1), and the observation value whose percentile is identical to the model percentile is the true or bias corrected value $X_{bcc}$. The bias correction is give as follows,

$X_{bcc}=F_{ob}^{-1}(F_{mc}\left( X_{mc} \right))$, (1)

where $F_{ob}$ and $F_{mc}$ are CDFs of observational data and current climate model data, respectively.

The information of model biases in the current climate data was applied to the future climate model results. There are also various choices of how to apply model biases to future climate results. In the present study, equidistant CDF matching method (EDCDFm) (Li 2010) is adapted to our future climate data. This method uses the relationship between correction amounts and percentiles. The relation was generated when performing bias correction of current climate results and used for the future climate results. This calculation is represented as follows,

$X_{bcf}=X_{mf}+F_{ob}^{-1}\left( F_{mf}\left( X_{mf} \right) \right)-F_{mc}^{-1}(F_{mf}\left( X_{mf} \right))$, (2)

where $F_{mf}$ is a CDF of future climate model data, and $X_{mf}$ and $X_{bcf}$ are a result of future climate model and its bias corrected value. Because only correction amounts are carried over from current climate results to future climate ones, the mean future changes are not changed by the bias correction method.

The actual procedure of the bias correction method is as follows,

(1) For each AMeDAS point and in each month, correction amount for daily temperature (shortwave radiation) as a function of the percentile was calculated using the CDFs of observational data and current climate model data. The nearest model grid point of the model to the AMeDAS point was used for the CDF calculation. Because empirical CDF was not smooth, several data were averaged to make 10 groups, each of which represented the mean values from *n* to *n*+10 percentile (n=0–9), and these 10 correction amounts were stored for each AMeDAS point and month. This grouping procedure makes the bias correction robust and reduces the harmful effect of outlier of the data.

(2) For each model grid point and in each month, the actual correction amount $\Delta X$ ($X_{bcc}-X_{mc}$ for current climate or $X_{bcf}-X_{mf}$ for future climate) is calculated as follows,

A$\frac{\sum_{i} {(1/r_{i})}^{2}\Delta X_{i}^{m}}{\sum_{i} {(1/r_{i})}^{2}}+(1-A)\frac{\sum_{i} {(1/r_{i})}^{2}\Delta X_{i}^{m+1}}{\sum_{i} {(1/r_{i})}^{2}}$, (3)

where indices *i* and *m* mean the AMeDAS index and month. The AMeDAS points whose distance from the target grid point was smaller than 42 km were used in the equation (3), and their weights for average were inversely proportional to the square of the distance between the AMeDAS and model points $r_{i}$. Two bias correction amounts $\Delta X_{i}^{m}$ and $\Delta X_{i}^{m+1}$ from adjacent two months were used, and the bias correction amount on each day was calculated by interpolating linearly between them.

(3) The correction amount calculated was added to the daily temperature (shortwave radiation) at each target model point. This procedure was adapted both for present and future climate data. In the present climate, the characteristics in the model grid point nearest to the AMeDAS point is similar to those of the AMeDAS point, but not identical because the information from other AMeDAS points surrounding the model grid point was taken into account.

(4) Annual-mean and 20-year mean temperature (shortwave radiation) was calculated using the bias-corrected daily-mean temperature (shortwave radiation).


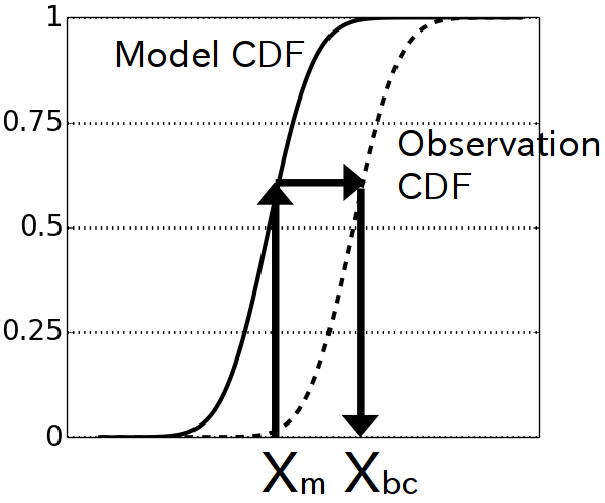


Fig. 1

Schematic figure of bias correction.

**References**

Li, Haibin, Justin Sheffield, and Eric F. Wood. "Bias correction of monthly precipitation and temperature fields from Intergovernmental Panel on Climate Change AR4 models using equidistant quantile matching." *Journal of Geophysical Research: Atmospheres* 115.D10 (2010).
