# Supplementary material for: Detecting latitudinal and altitudinal expansion of invasive bamboo Phyllostachys edulis and Phyllostachys bambusoides (Poaceae) in Japan to project potential habitats under 1.5°C–4.0°C global warming
Source: Ecol Evol. 2017 Oct 18;7(23):9848–59. doi: 10.1002/ece3.3471 (PMC5723622; doi:10.1002/ece3.3471)
Supplement: Supplementary file 7 [file ECE3-7-9848-s007.pdf]

Supporting Information Table S1. Effect size of selected explanatory variables, model fit, confusion matrix, and predictability indices of the best-10 min

	Effect size (z-value) of explanatory variable[*1]										
Order	Mean annual temperature	Maximum temperature or a year	Minimum temperature or a year	Warmth Index (WI)	Coldness Index (CI)	Annual precipi-tation	Precipitation in growing season	Precipitation in non-growing season	Sun radiation	Ratio of farmland area	Ratio of forest area
1	-	-	-	5.111***	-	-	-	-	2.165*	-	-
2	-	5.395***	-	-	-	0.938	-	-	2.093*	-	-
3	-	5.503***	-	-	-	-	-	-	1.961*	-	-
4	4.968***	-	-	-	-	-	-	-	2.166*	-	-
5	-	-	-	4.831***	-	-	-	-	2.238*	-	0.795
6	-	-	-	5.046***	-	-	0.741	-	2.224*	-	-
7	-	5.395***	-	-	-	0.938	-	-	2.093*	-	-
8	-	5.009***	-	-	-	-	1.621	-	1.571	-	-0.950
9	4.868***	-	-	-	-	-	0.902	-	2.256*	-	-
10	-	5.256***	-	-	-	-	1.620	-0.904	1.624	-	-

[*1] -: not selected, ***: *P*-value<0.001, *: <0.5

[*2] the area under the ROC curve (Swets, 1973)

[*3] Matthews Correlation Coefficient (Matthews, 1975)

[*4] Powers (2011)

[*5] Positive predictive value

[*6] Negative predictive value

imum-AICc GLMs.

Model fit				Confusion matrix				Threshold	Predictability index							
degree of freedom	log Likelihood	AICc	delta AICc	True positive	False positive	False negative	True negative		AUC[*2]	MCC[*3]	Informed-ness[*4]	Accuracy	Sensi-tivity	Speci-ficity	PPV[*5]	NPV[*6]
3	-32.907	71.984	0	109	7	5	24	0.660	0.927	0.749	0.767	0.917	0.940	0.828	0.956	0.774
4	-32.121	72.527	0.543	103	13	3	26	0.829	0.933	0.708	0.784	0.890	0.888	0.897	0.972	0.667
3	-33.218	72.607	0.623	105	11	3	26	0.823	0.939	0.736	0.802	0.903	0.905	0.897	0.972	0.703
3	-33.226	72.622	0.637	112	4	6	23	0.606	0.922	0.780	0.759	0.931	0.966	0.793	0.949	0.852
4	-32.579	73.443	1.459	102	14	3	26	0.794	0.923	0.694	0.776	0.883	0.879	0.897	0.971	0.650
4	-32.618	73.522	1.538	109	7	5	24	0.636	0.921	0.749	0.767	0.917	0.940	0.828	0.956	0.774
4	-32.735	73.756	1.772	103	13	3	26	0.829	0.933	0.708	0.784	0.890	0.888	0.897	0.972	0.667
5	-31.672	73.776	1.792	105	11	3	26	0.807	0.927	0.736	0.802	0.903	0.905	0.897	0.972	0.703
4	-32.789	73.863	1.879	107	9	5	24	0.702	0.920	0.716	0.750	0.903	0.922	0.828	0.955	0.727
5	-31.734	73.900	1.916	105	11	4	25	0.758	0.934	0.710	0.767	0.897	0.905	0.862	0.963	0.694
