# Supplementary material for: Detecting latitudinal and altitudinal expansion of invasive bamboo Phyllostachys edulis and Phyllostachys bambusoides (Poaceae) in Japan to project potential habitats under 1.5°C–4.0°C global warming
Source: Ecol Evol. 2017 Oct 18;7(23):9848–59. doi: 10.1002/ece3.3471 (PMC5723622; doi:10.1002/ece3.3471)
Supplement: Supplementary file 8 [file ECE3-7-9848-s008.pdf]

Supporting Information Table S2. Predictability of different combinations of modelling methods and explanatory variables.

Modelling method	Expression of explanatory valiables in model fomula[*1]	Confusion matrix				Threshold	Predictability index							
		True positive	False positive	False negative	True negative		AUC[*2]	MCC[*3]	Informed-ness[*4]	Accuracy	Sensi-tivity	Speci-ficity	PPV[*5]	NPV[*6]
Generalized linear model	tmp	112	4	7	22	0.581	0.911	0.755	0.724	0.924	0.966	0.759	0.941	0.846
Generalized additive model	tmp	111	5	7	22	0.608	0.882	0.735	0.716	0.917	0.957	0.759	0.941	0.815
Random Forest	tmp	114	2	8	21	0.213	0.891	0.774	0.707	0.931	0.983	0.724	0.934	0.913
Boosted Regression Trees	tmp	113	3	7	22	0.784	0.860	0.776	0.733	0.931	0.974	0.759	0.942	0.880
Generalized linear model	tmp+radiation	112	4	6	23	0.606	0.922	0.780	0.759	0.931	0.966	0.793	0.949	0.852
Generalized additive model	s(tmp)+s(radiation)	108	8	6	23	0.687	0.903	0.707	0.724	0.903	0.931	0.793	0.947	0.742
Random Forest	tmp+radiation	113	3	7	22	0.729	0.850	0.776	0.733	0.931	0.974	0.759	0.942	0.880
Boosted Regression Trees	tmp+radiation	113	3	7	22	0.785	0.846	0.776	0.733	0.931	0.974	0.759	0.942	0.880
Generalized linear model	tmp+radiation+pre.grow	107	9	5	24	0.702	0.920	0.716	0.750	0.903	0.922	0.828	0.955	0.727
Generalized additive model	s(tmp)+s(radiation)+s(pre.grow)	113	3	7	22	0.527	0.917	0.776	0.733	0.931	0.974	0.759	0.942	0.880
Random Forest	tmp+radiation+pre.grow	113	3	7	22	0.618	0.891	0.776	0.733	0.931	0.974	0.759	0.942	0.880
Boosted Regression Trees	tmp+radiation+pre.grow	113	3	7	22	0.791	0.840	0.776	0.733	0.931	0.974	0.759	0.942	0.880
Generalized linear model	tmp+radiation+pre.grow+forest+forest^2+farmland+farmland^2	100	16	3	26	0.775	0.913	0.669	0.759	0.869	0.862	0.897	0.971	0.619
Generalized additive model	s(tmp)+s(radiation)+s(pre.grow)+s(forest)+s(farmland)	109	7	6	23	0.737	0.872	0.724	0.733	0.910	0.940	0.793	0.948	0.767
Random Forest	tmp+radiation+pre.grow+forest+farmland	113	3	7	22	0.665	0.896	0.776	0.733	0.931	0.974	0.759	0.942	0.880
Boosted Regression Trees	tmp+radiation+pre.grow+forest+farmland	103	13	5	24	0.795	0.829	0.657	0.716	0.876	0.888	0.828	0.954	0.649

[*1] tmp: mean annual temperature, radiation: sun radiation, pre.grow: precipitation in growing season (>=5°C), forest: ratio of forest in 1 km radius, farmland: ratio of farmland, s: spline

[*2] the area under the ROC curve (Swets, 1973)

[*3] Matthews Correlation Coefficient (Matthews, 1975)

[*4] Powers (2011)

[*5] Positive predictive value

[*6] Negative predictive value
