# Supplementary material for: Detecting latitudinal and altitudinal expansion of invasive bamboo Phyllostachys edulis and Phyllostachys bambusoides (Poaceae) in Japan to project potential habitats under 1.5°C–4.0°C global warming
Source: Ecol Evol. 2017 Oct 18;7(23):9848–59. doi: 10.1002/ece3.3471 (PMC5723622; doi:10.1002/ece3.3471)
Supplement: Supplementary file 9 [file ECE3-7-9848-s009.pdf]

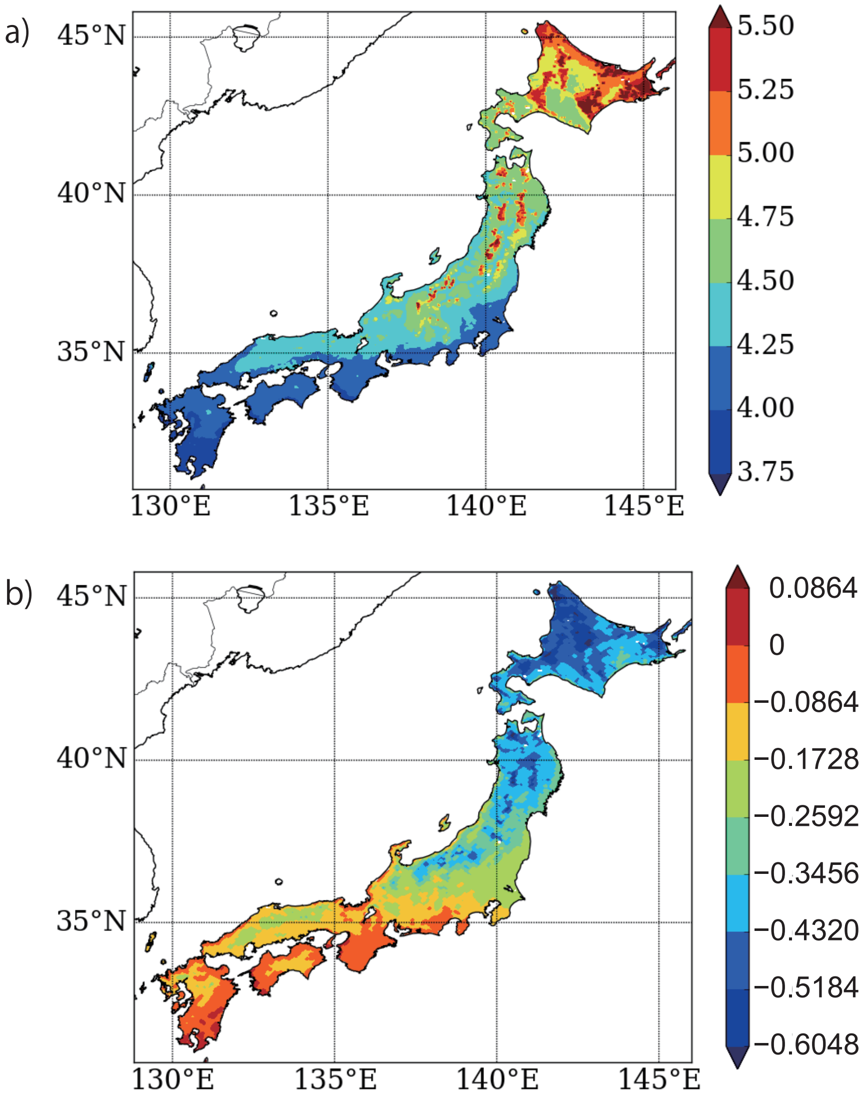

Supporting Information fig S5. Geographical distribution of future changes in a) mean temperature (°C) and b) shortwave radiation (MJ/m2/day), which is the differences of annual and over 20-simulation-years averages between current (1980–2000) and future (2076–2096) experiments. Mean annual temperature was projected to increase by 4.45°C averaged over Japan land area between the periods of 1980–2000 and 2076–2096 (a). This exceeds an increment in the global average (3.49°C) and approximately equals to that in global land area average (4.56°C). The change in annual-mean shortwave radiation was −0.25 MJ/m2/day averaged over Japan land area, but the signs of the change take both positive and negative depending on locations (b). Globally seen, this change is −0.30 MJ/m2/day (whole world) and −0.16 MJ/m2/day (land area). Although the general trend exists that the future change of temperature (solar radiation) increases (decreases) with latitudes, the changes depended on locations, particularly in complex terrain. The high resolution of 5km enabled to reproduce these spatially detailed patterns.
