# Supplementary material for: Detecting latitudinal and altitudinal expansion of invasive bamboo Phyllostachys edulis and Phyllostachys bambusoides (Poaceae) in Japan to project potential habitats under 1.5°C–4.0°C global warming
Source: Ecol Evol. 2017 Oct 18;7(23):9848–59. doi: 10.1002/ece3.3471 (PMC5723622; doi:10.1002/ece3.3471)
Supplement: Supplementary file 11 [file ECE3-7-9848-s011.docx]

Data S3. Dissimilarity index: Modified version of the multivariate environmental similarity surface (MESS, after Elith et al. 2010)

MESS calculation represents how similar a point is to a reference set of points, with respect to a set of predictor variables (*V_1_*, *V_2_*, ...). Elith et al. (2010) allows negative values – these will be sites where at least one variable has a value that is outside the range of environments over the reference set, so these are novel environments. The values in the MESS are influenced by the full distribution of the reference points, so that sites within the environmental range of the reference points but in relatively unusual environments will have a smaller value than those in very common environments.

The MES of a point *P* is calculated as follows (Elith et al. 2010):

1. Let min*_i_* be the minimum value of variable *V_i_* over the reference point set, and similarly for max*_i_*.

2. Let p*_i_* be the value of variable *V_i_* at point *P*.

3. Let f*_i_* be the percent of reference points whose value of variable *V_i_* is smaller than p*_i_*.

4. Then the similarity of *P* with respect to variable *V_i_* is:

(p*_i_* - min*_i_*) / (max*_i_* - min*_i_*) * 100 if f*_i_* = 0

2 * f*_i_* if 0 < f*_i_* ≤ 50

2 * (100 - f*_i_*) if 50 ≤ f*_i_* < 100

(max*_i_* - p*_i_*) / (max*_i_* - min*_i_*) * 100 if f*_i_* = 100

5. Finally, the multivariate similarity of *P* is the minimum of its similarity with respect to each variable.

Because MESS does not show direction of change (i.e. increase or decrease) in a variable, we modified the steps 4 and 5 as follows.

4’. The dissimilarity of *P* with respect to variable *V_i_* is:

(p*_i_* - min*_i_*) / (max*_i_* - min*_i_*) * 100 – 100 if f*_i_* = 0

2 * f*_i_* - 100 if 0 < f*_i_* ≤ 50

-1 * 2 * (100 - f*_i_*) + 100 if 50 ≤ f*_i_* < 100

-1 * (max*_i_* - p*_i_*) / (max*_i_* - min*_i_*) * 100 + 100 if f*_i_* = 100

The values of dissimilarity under -100 or over 100 represent novel environments (toward decreasing and increasing trends, respectively).

5’. Finally, the multivariate dissimilarity of *P* is the absolute maximum of its dissimilarity with respect to each variable.


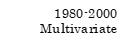

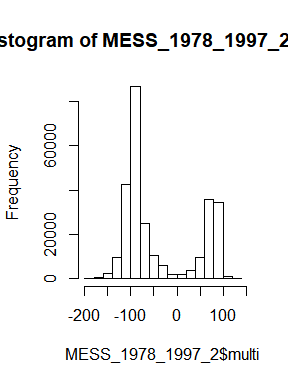

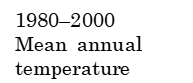

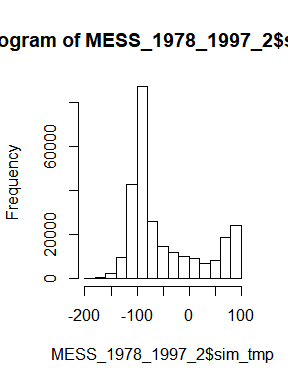

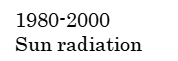

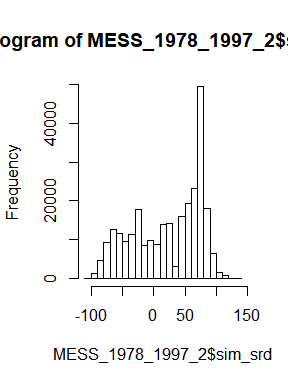

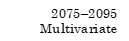

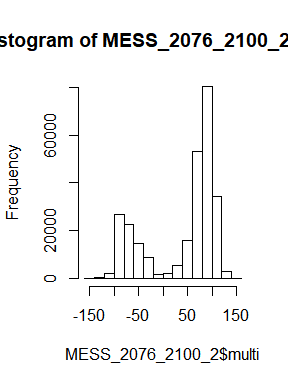

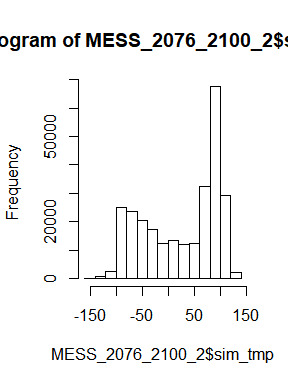

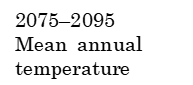

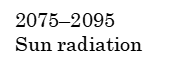

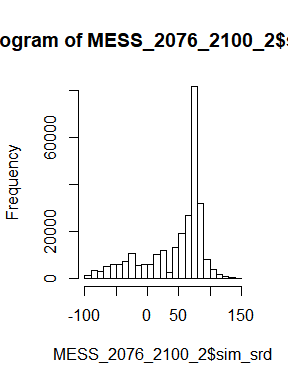


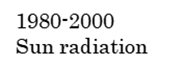


Extrapolation

to new

climate

Range of

reference

points used

for modelling

Extrapolation

Fig. Modified version of the multivariate environmental similarity surface (MESS, Elith et al. 2010), or dissimilarity, showing the extent of extrapolation from the reference points (i.e. 145 AMeDAS stations in 2002–2011) for current (1980–2000, upper panels) and future (2075–2095, lower) climates. “Multivariate” represents absolute maximum between mean annual temperature and sun radiation. A histogram shows distribution of dissimilarity calculated for each grid cell in the focal area.

In current temperature (upper left), rather many areas with high altitude and latitude showed extrapolation with lower temperature (values < -100), which is also reflected in multivariate surface (upper right). In future temperature (lower left), most of area was included in the range of reference points except for area with higher and lower extreme of altitude and latitude. Overall, the extent of extrapolation seems modest in future prediction, whereas extrapolation for lower temperature was observed in current prediction.
