# Supplementary material for: Detecting latitudinal and altitudinal expansion of invasive bamboo Phyllostachys edulis and Phyllostachys bambusoides (Poaceae) in Japan to project potential habitats under 1.5°C–4.0°C global warming
Source: Ecol Evol. 2017 Oct 18;7(23):9848–59. doi: 10.1002/ece3.3471 (PMC5723622; doi:10.1002/ece3.3471)
Supplement: Supplementary file 12 [file ECE3-7-9848-s012.pdf]

(1) Spatial pattern in 20-year mean temperature difference δT(x,y)

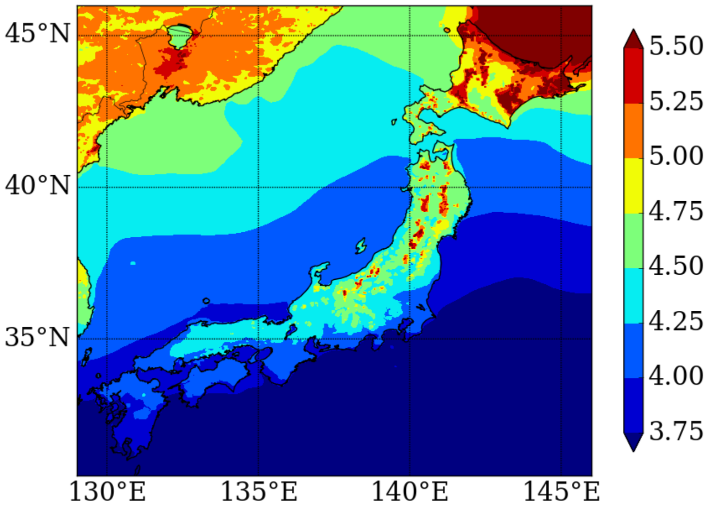

(2) Scaling pattern

δT(x,y) / Global mean future temperature increase in our model

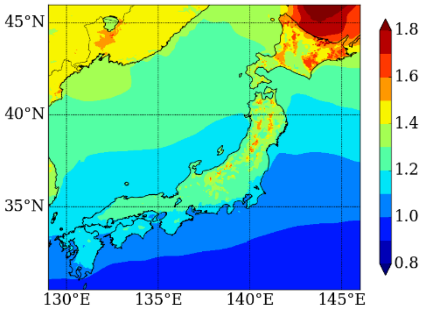

Case of ensemble mean SST

(3) Calculate the progress of global warming using CMIP5 data

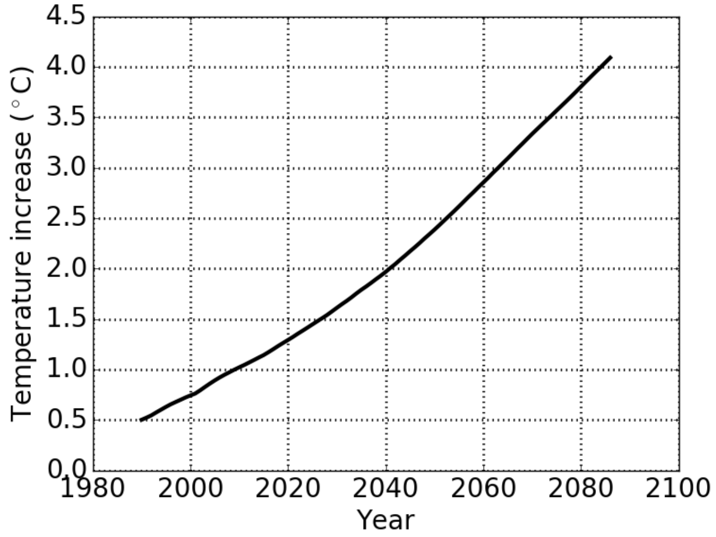

(4) Inflation of scaling pattern according to the progress of global warming (X℃-0.5℃), and is added to the present climate result.

Scaling pattern

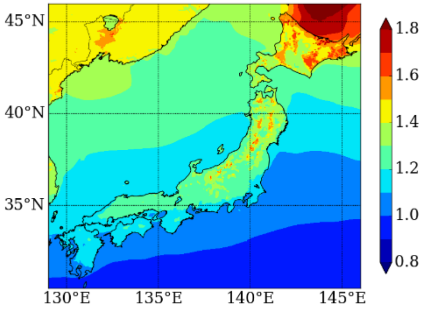

×

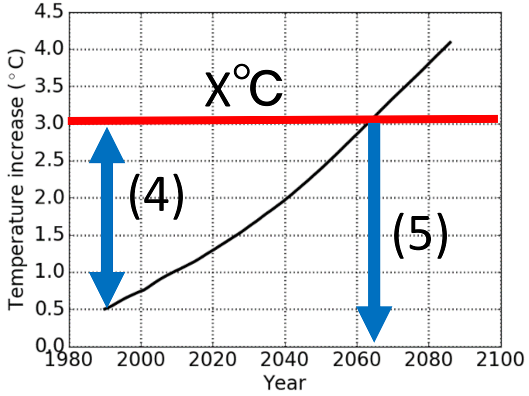

+

Present temperature

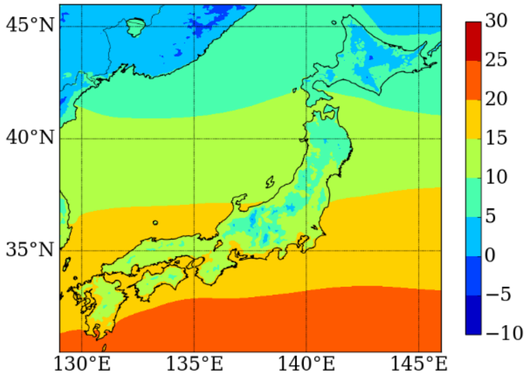

(5) The year when the global warming exceeds a specified level is calculated with (3)
