# Supplementary material for: Detecting latitudinal and altitudinal expansion of invasive bamboo Phyllostachys edulis and Phyllostachys bambusoides (Poaceae) in Japan to project potential habitats under 1.5°C–4.0°C global warming
Source: Ecol Evol. 2017 Oct 18;7(23):9848–59. doi: 10.1002/ece3.3471 (PMC5723622; doi:10.1002/ece3.3471)
Supplement: Supplementary file 6 [file ECE3-7-9848-s006.pdf]

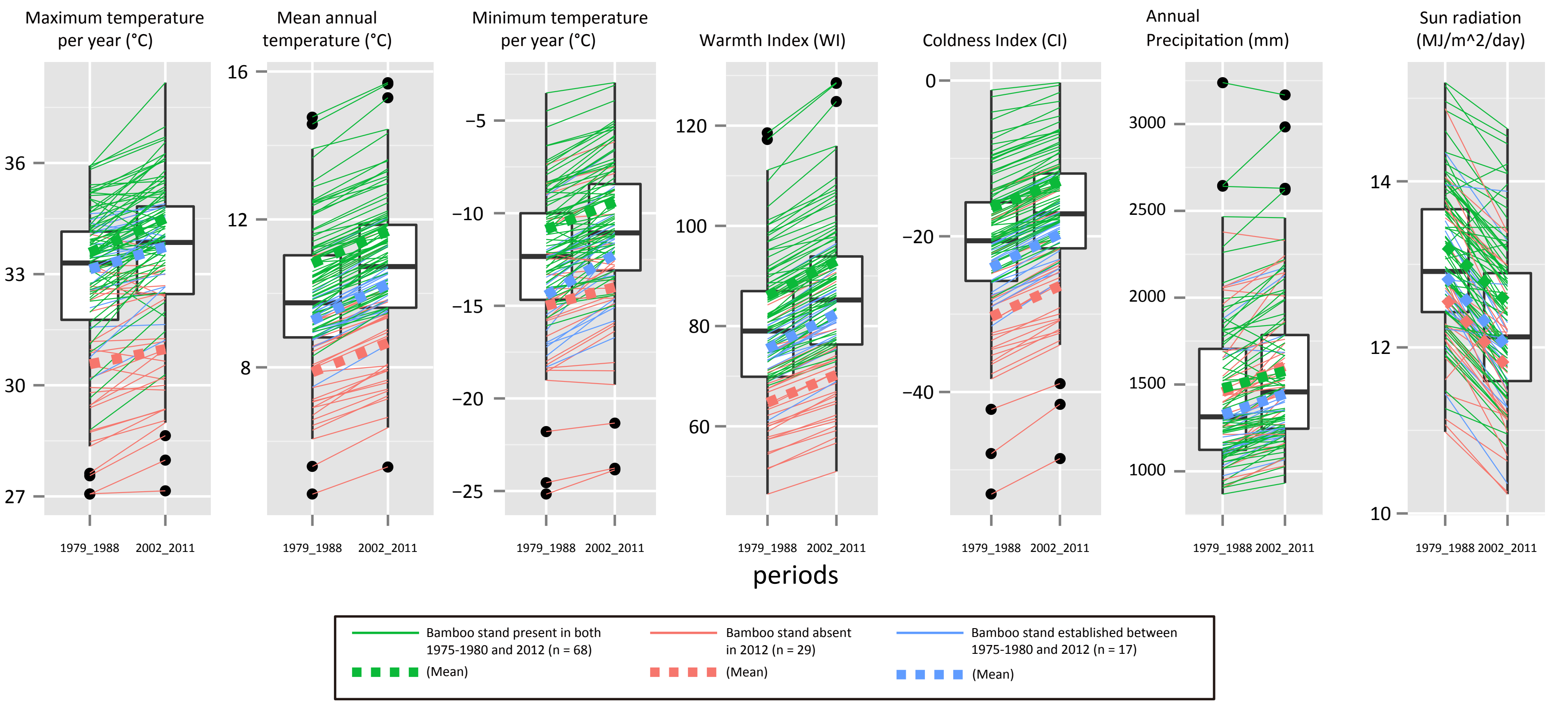


Supporting Information fig S4. Changes in the means of climatic variables between 1979–1988 and 2002–2011. Boxplot includes all meteorological stations analysed (n = 114, we excluded 24 sites where bamboo stands were present in 2012 but unknown for 1975–1980). Solid lines connecting the different periods correspond to each meteorological station. Colours represent different categories of bamboo distribution in the study sites: red, absent in 2012; green, present in both 1975–1980 and in 2012; blue, established during 1975–1980 and 2012. Dotted lines represent the mean of each category.
